# Applications of mesenchymal stem cell-exosome components in wound infection healing: new insights

**DOI:** 10.1093/burnst/tkae021

**Published:** 2024-08-13

**Authors:** Arshia Fakouri, Zahra-Sadat Razavi, Adil Tawfeeq Mohammed, Abbas Hameed Abdul Hussein, Hamed Afkhami, Mohammad Hosseini Hooshiar

**Affiliations:** Student Research Committee, USERN Office, Lorestan University of Medical Sciences, Khorramabad 6813833946, Iran; Physiology Research Center, Iran University of Medical Sciences, Tehran, Iran; Al-Noor University College, Nineveh, Iraq; Ahl Al Bayt University, Karbala, Iraq; Nervous System Stem Cells Research Center, Semnan University of Medical Sciences, Semnan, Iran; Cellular and Molecular Research Center, Qom University of Medical Sciences, Qom, Iran; Department of Medical Microbiology, Faculty of Medicine, Shahed University, Tehran, Iran; School of Dentistry, Tehran University of Medical Sciences, Tehran, Iran

**Keywords:** Mesenchymal stem cells, Exosome component, mesenchymal stem cell-exosome, Wound, Wound healing, Wound infection

## Abstract

The healing process at a wound is made up of many types of cells, growth factors, the extracellular matrix, nerves and blood vessels all interacting with each other in complex and changing ways. Microbial colonization and proliferation are possible at the place of injury, which makes infection more likely. Because of this, any cut has a chance of getting an infection. Researchers have found that wound infections make patients more upset and cost the healthcare system a lot of money. Surgical site infections happen a lot to people who have recently had surgery. This study shows that such surgical infection is linked to a high rate of illness and death. This is shown by the fact that 25% of patients get serious sepsis and need to be transferred to an intensive care unit. In both animal models and people, mesenchymal stem cells (MSCs) play an active role in all stages of wound healing and have positive effects. Exosomes are one of the main things MSCs release. They have effects that are similar to those of the parent MSCs. Various effector proteins, messenger RNA and microRNAs can be transported by extracellular vesicles to control the activity of target cells. This has a big impact on the healing process. These results suggest that using MSC-exosomes as a new type of cell-free therapy could be a better and safer option than whole cell therapy. This review is mostly about how to use parts of MSC-exosomes to help wound infections heal.

## Graphical Abstract

**Graphical Abstract:**
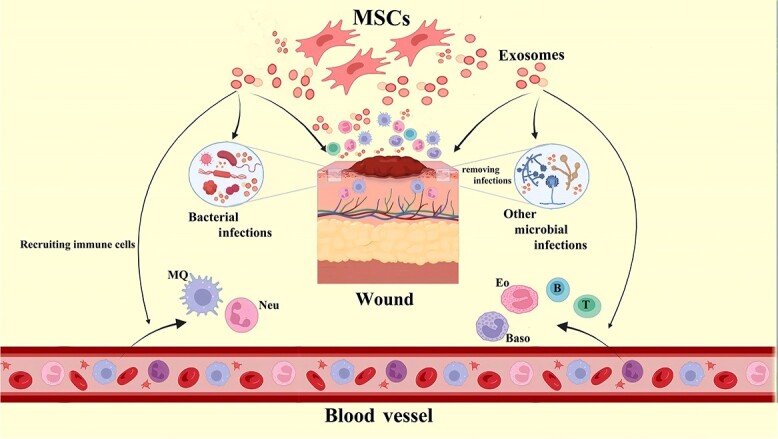

Highlights

- MSC-derived exosomes significantly reduced oxidative stress and protein misfolding in myocardial tissues during ischemia/reperfusion injury, highlighting their protective effects.
- Treatment with MSC-derived exosomes led to a notable decrease in inflammatory markers and improved tissue repair in models of burn injuries and dilated cardiomyopathy.
- Application of ADSC-Exos in cutaneous wound healing models resulted in accelerated wound closure, enhanced skin regeneration, and reduced chronic inflammation.

## Background

The integumentary system is comprised of the largest sensory organ in the human body, which is the skin. Furthermore, it serves as a tangible obstacle and safeguards against potential harm caused by hazardous substances or pathogenic microorganisms [1]. Mechanical injuries, such as cuts or burns, can compromise skin integrity, leading to exposure of subcutaneous tissue to the external environment. The uncovered tissue creates a favorable habitat for pathogenic microorganisms to flourish, due to the presence of warmth, moisture and nutrients [2]. Therefore, the area of injury becomes susceptible to microbial colonization and proliferation, and any wound carries a certain degree of susceptibility to infection. The occurrence of wound infection results in heightened patient trauma and imposes financial strain on the healthcare system, as evidenced by previous research [3]. Surgical site infections are a common occurrence among post-operative surgical patients [4]. At present surgical infection exhibits a significant association with elevated morbidity and mortality rates, whereby a quarter of patients are prone to developing severe sepsis necessitating their transfer to an intensive care unit [5].

Stem cell-based therapies have demonstrated significant potential for the regeneration of damaged tissues in both preclinical and clinical trials, as evidenced by recent research [6]. Stem cell-based remedies offer several advantages over conventional therapies that rely on growth factors or cytokine biologicals. This is due to the superior regenerative capacity of stem cells, which can promote healing and regeneration through multifactorial mechanisms. Mesenchymal stem cells (MSCs) have been identified as the predominant stem cell category that has demonstrated clear therapeutic benefits in addressing a range of tissue injuries [7]. MSCs are multipotent because they can self-renew and differentiate into multiple cell types. Stem cells are abundant in the human body and can be extracted from a wide variety of tissues, such as bone marrow, adipose tissue, tooth tissue and the umbilical cord [8].

The administration of MSC therapy has been observed to alleviate bacterial clearance by enhancing the migratory and phagocytic capabilities of neutrophils, which is achieved through the upregulation of cytokines such as interleukin 6 (IL-6), IL-8 and granulocyte-macrophage colony-stimulating factor. These molecules ultimately contribute to the elimination of the infection and aid in the process of tissue restoration, as demonstrated in the phenomenon of wound healing [9].

Numerous academic studies have investigated the immunomodulatory characteristics and suggested mechanisms of MSCs. The immunomodulatory properties of the local microenvironment and the capacity to attract reparative cells and migrate to injured tissue are among the attributes that have piqued interest in the utilization of MSCs for cutaneous wound healing. This interest has arisen due to the absence of definitive remedies for persistent wounds in the last 10 years [10]. The therapeutic approach of MSCs for skin wounds involves restoration and substitution of cellular substrates, reduction of inflammation, promotion of angiogenesis and the facilitation of reparative cell migration. However, the intricate mechanisms of MSC differentiation, mobilization and homing are still not fully understood and require additional investigation [2,11,12].

Exosomes are a type of lipid bilayer vesicle that exhibit a spherical shape and possess a range of diameters spanning from 30–150 nm. Exosomes are generated through a sequence of membrane-trafficking events [13]. Early endosomes might originate from the invagination of the plasma membrane or from the budding of membranes from intracellular organelles. Multivesicular bodies (MVBs) are formed when early endosomes undergo invagination and simultaneously generate intraluminal vesicles (ILVs). MVBs contain ILVs, which can go in one of two directions. Both lysosomal degradation and exocytosis during MVB transport culminate in membrane fusion with the plasma membrane. ILVs are secreted into the extracellular environment, where they give rise to exosomes [14]. Exosomes that have been released may reach their target cells through paracrine signaling or circulation, and subsequently undergo internalization by the recipient cells. This process can occur through various mechanisms, including ligand–receptor interaction, surface molecule-mediated endocytosis, micropinocytosis, phagocytosis or plasmatic membrane fusion with the recipient cells [15]. Upon the discharge of exosome-encapsulated cargo into the cytoplasm of the recipient, modifications of intracellular signaling pathways transpire in the recipient cells, thereby regulating cellular processes and functions [3].

The potential of MSC-exosome components in wound infection healing has attracted considerable attention in recent years, with the possibility of transforming the field of wound care. As experts in the field continue to explore new approaches to tackle the issues related to wound infections, the utilization of exosomes derived from MSCs has emerged as a hopeful therapeutic strategy. These tiny extracellular vesicles, released by MSCs, have a crucial function in communicating between cells and are recognized for transporting a wide variety of active substances, such as proteins, lipids and nucleic acids [16]. The distinct composition of MSC-exosomes allows them to influence different cellular processes related to wound healing, including inflammation, angiogenesis and tissue regeneration. In addition, the capacity to transmit genetic information to other cells amplifies their potential as valuable therapeutic agents. This paper seeks to offer fresh perspectives on the uses of MSC-exosome components in the healing of wound infections [17]. It accomplishes this by examining existing literature on their mechanisms of action, preclinical and clinical studies and future prospects in this area. Through a thorough analysis of the existing evidence, this paper aims to add to the expanding knowledge in the field of MSC-exosomes for wound care. This research has the potential to lead to the creation of improved and tailored therapeutic methods [18].

Receptors, enzymes, transcription factors, lipids, extracellular matrix (ECM) proteins, and Mitochondrial DNA (mtDNA), Single-stranded DNA (ssDNA), Double-Stranded DNA (dsDNA), messenger RNA (mRNA) and microRNA (miRNA) are only some of the cytoplasmic molecules and membrane proteins found in MSC exosomes [19]. The contents of exosomes can have an effect on the proliferation, migration, death and immunomodulation of the recipient cells. By manipulating important signaling pathways such PI3Ks/AKT, JAK/STAT, TGF-/Smad and Wnt/−catenin, regeneration processes in target organs can be altered [20]. Several functions of MSC exosomes have been identified, including regulation of macrophage activation, initiation of angiogenesis, stimulation of keratinocyte and dermal fibroblast proliferation and migration, and modulation of myofibroblasts’ innate capacity to change ECM turnover [21].

Exosomes-based therapies have the potential to offer advantages over conventional cell-based therapies by circumventing the adverse effects associated with transplanted cells, such as immune rejection [22]. MSCs may be able to significantly influence inflammation reduction via the exosomes they release by suppressing M1 polarization and enhancing M2 polarization. The enhancement of skin wound healing may be facilitated by the regulation of M2 polarization through exosomes derived from MSCs, as indicated by previous research [23].

## Review

### Wound and wound infection

As a result of damage caused by blunt force or compression, the skin may become lacerated or perforated (open wounds), or it may bruise (closed wounds). In the study of pathology, the term “wound” refers to any injury that occurs to the epidermis, which is the topmost layer of skin [24,25]. The series of steps that take place while the body attempts to mend itself after suffering an injury is referred to as the “wound healing process”. A wound’s classification depends on the degree of contamination it has received. A clean wound is one that has been created in a sterile environment, free of any bacteria or other pathogens, and is therefore more likely to heal without incident [23]. A contaminated wound is one that contains pathogenic organisms and foreign substances and has most likely resulted from an unintentional injury [26]. Clinical indications of infection manifest in a wound when pathogenic organisms are present and proliferating (yellow appearance, soreness, redness, oozing pus). A colonized wound is a chronic condition that makes it hard to heal because it contains harmful organisms (e.g. bedsore) [27]. The most common causative organisms associated with wound infections include *Staphylococcus aureus/MRSA*, *Streptococcus pyogenes*, *Enterococci* and *Pseudomonas aeruginosa* [28].

The overall course of therapy is determined by the nature, origin and severity of the wound, as well as the involvement of tissues other than the skin (dermis) [29]. Most lacerations need to be examined, cleaned and bandaged as soon as possible after they happen [30]. Bruises and other minor wounds heal on their own, and any darkening to the skin should fade away within a week or two. Abrasions are defined as wounds with unbroken skin (non-penetration through dermis to subcutaneous fat) and can be treated simply by keeping the affected region clean, first with soap and water [31]. Infection risk increases with the depth of a puncture wound. To prevent infection, the puncture incision is left exposed at its point of entrance [32,33].

Skin healing is a highly structured physiological process. Inflammation, proliferation and maturation are three reciprocally overlapping stages of cell interaction [34,35]. This mechanism involves endogenous cutaneous MSCs such dermal papilla cells and dermal sheath cells. Perivascular pericytes and adipose tissue MSCs may function as MSCs *in vivo* [23]. Most trauma instances, such as severe burns and persistent ulcers, lack dermal tissue, therefore endogenous cutaneous MSCs cannot participate in wound self-repair. Hence, exogenous MSCs are used to repair wounds. MSCs improve wound healing and scarring regardless of their source. MSCs are interesting therapy alternatives in regenerative medicine and have rapidly developed as a cell therapeutic tool for wound repair over the past decade because of their extensive differentiation capacity [36]. A few clinical studies involving bone marrow-derived MSCs (BMSCs) and other tissue-of-origin MSCs employed in animal models provide much of the data for MSCs’ wound healing potential [26]. These therapeutic trials transplant autologous BMSCs. Bone marrow aspiration, a safe but painful and intrusive operation, can cause infection and bleeding. Bone marrow cells also decrease with ageing [37]. Therefore, various alternatives such as adipose-derived stem cells (ADSCs), cutaneous MSCs, amniotic fluid and umbilical cord MSCs have been utilized [38]. ADSCs and dermal MSCs are abundantly present in adipose and skin tissues and can be extracted with minimal invasiveness. Moreover, they do not pose any ethical concerns, rendering them appropriate substitutes for BMSCs. ADSCs and dermal MSCs have been found to possess immunogenic properties and the ability to differentiate into BMSCs [39,40]. ADSCs have been used to treat burns and ulcers in clinical studies [41]. MSCs were found to enhance wound healing in clinical studies [37,42]. To date, there is a lack of clinical studies investigating the efficacy of human amniotic membrane-derived mesenchymal stem cells or human umbilical cord-derived mesenchymal stem cells in the context of wound healing.

The present clinical investigations provide positive indications that treatments based on MSCs are safe and potentially efficacious. Furthermore, there is no discernible evidence to suggest that any particular MSC tissue origin is superior in terms of promoting wound healing [43]. From a biological standpoint, it has been demonstrated that MSC-exosomes bear a striking resemblance to whole MSCs, indicating that they represent the primary paracrine element of MSCs [42]. Furthermore, due to the direct interaction of MSC-exosomes with recipient cells, they exhibit potent physiological impacts, thereby distinguishing them from MSCs. Furthermore, it has been observed that MSC-exosomes possess a greater capacity for preservation and transportation over extended periods of time at a temperature of 70°C. This is attributed to the protective nature of the exosome’s plasma membrane, which impedes the degradation of its active constituents. Finally, it can be observed that managing variables such as dosage, administration method and timing is effortless [44,45].

Exosomes derived from MSCs have demonstrated potential in the healing of wounds. These exosomes possess the remarkable capacity to enhance damage repair, facilitate tissue regeneration and expedite wound healing. They have the ability to promote the healing of skin wounds, stimulate the growth and movement of both normal and chronic wounds, and improve the formation of new blood vessels. Exosomes derived from MSCs have shown promising results in promoting wound healing in various organs, including the skin and epidermal wounds [46]. They have been discovered to have therapeutic properties in a range of diseases, such as wound repair, and are considered as potential agents for nano-therapeutics. Studies have indicated that treatments utilizing exosomes have been found to enhance the healing process of wounds. This is achieved by promoting the growth of new blood vessels, facilitating the regeneration of skin cells and encouraging the formation of collagen, all while minimizing the development of scars. However, more research is necessary to gain a complete understanding of their treatment mechanism, effective extraction and separation methods, and standardized production, before they can be widely applied in clinical settings. In the realm of wound healing and regenerative medicine, the potential of MSC-exosomes is truly remarkable. Ongoing research is poised to amplify their clinical application even more [47].

#### Exosome biogenesis

In 1983, Stahl *et al*. identified multiple exosomes during the maturation of mammalian reticulocytes, which are immature red blood cells [48]. Johnstone and colleagues provided the initial description of exosomes in 1987. Erythrocytes, commonly known as red blood cells, are derived from precursor cells known as reticulocytes [49]. During the course of this procedure, exosomes demonstrate a degree of specificity in eliminating particular proteins from the plasma membrane of reticulocytes [50]. Similar to other cells found in mammals, reticulocytes regularly undergo the process of endocytosis, whereby their plasma membranes are internalized to form endosomes. In contrast, alternative varieties of endosomes internalize minuscule vesicles containing fragments of their membranes at a frequency of once per hour [51]. The larger endosomes that contain numerous smaller vesicles are commonly known as MVBs [52]. The MVBs undergo fusion with the cell membrane, thereby facilitating the release of intracellular vesicles into the extracellular milieu. These vesicles are commonly referred to as exosomes [53].

Exosomes, which are membrane-bound extracellular vesicles, are present in the endosomal compartment of the majority of eukaryotic cells. MVBs are a type of endosome that contain ILVs which extend into the lumen of the endosome [54]. The ILVs are secreted as exosomes subsequent to their amalgamation with the cellular exterior, specifically the plasma membrane [49,55].

Blood, urine and cerebrospinal fluid are only a few of the biological fluids that include exosomes and other extracellular vehicles (EVs). Exosomes have also been seen to be present in tissue matrixes; these exosomes are referred to as matrix-bound nanovesicles [56,57]. A cell that was cultured has been observed to secrete exosomes into the surrounding growth medium. Exosomes are typically smaller than other EVs as a result of the size limitation imposed by their originating MVBs. Their size varies between 30 and 150 nm, which is comparable to that of numerous lipoproteins and significantly smaller than cells [7,58].

#### Exosome components

The average size of exosomes, a form of EV, is between 40 and 150 nm. Lipids, proteins, RNAs [mRNAs, tRNAs, long noncoding RNAs (lncRNAs), mtDNA, miRNAs] and lipid phosphate esters are only some of the bioactive compounds found in these cellular components [59]. Apart from certain generic molecules, they also encompass distinct proteins specific to tissue types, which are indicative of their respective origins. Numerous cell types, including immune cells, tumor cells and MSCs, have been shown to secrete exosomes, and this secretion has been quantitatively confirmed. MSC exosomes include a wide variety of chemicals, such as mRNAs, miRNAs, cytokines and growth factors [60]. There is evidence that exosomes can transport miRNAs to induce M2 macrophage polarization. He *et al*. [61] revealed that exosomes produced from bone marrow MSCs caused polarization of macrophages toward the M2 phenotype; they also reported that miR-223, originating from MSC exosomes and targeting pknox1, controlled the polarization. The results of the study demonstrated that miR-181c could successfully inhibit the toll-like receptor 4 (TLR4) signaling pathway, maintaining the elevated levels of TNF-α and IL-1β as well as the reduced levels of IL-10 in macrophages, which is indicative of M2 polarization. In particular, lipopolysaccharide (LPS) preconditioning can improve the polarization effects of MSC exosomes. LPS pre-exosomes, or exosomes generated from MSCs that have been pre-treated with LPS to reduce inflammation associated with wound healing, have been shown to have more immunotherapeutic potential and activation of M2 macrophages than untreated MSC-derived exosomes [62]. The distinct expression of let-7b in LPS pre-exosomes and the let-7b/TLR4/NFκB/STAT3/AKT regulatory signaling pathway in macrophages are linked to the heightened impact. Furthermore, it has been demonstrated that adipocyte-derived MSC (ADMSC) exosomes have comparable effects on macrophage polarization. ADMSC-exosomes were found to have beneficial effects on fibroblasts in Zhang *et al*.’s study [63]. These effects included promoting collagen deposition and the expression of growth factors, including transforming growth factor-β1 (TGF-β1) and basic fibroblast growth factor (bFGF), both *in vitro* and *in vivo*, by modulating the PI3K/AKT signaling pathway. In addition to fibroblasts, BMSC-derived exosomes have the ability to inhibit the miR-93-3p/APAF1 axis, which is responsible for apoptosis in human immortalized epidermal cells when exposed to hydrogen peroxide [64]. Additionally, studies have shown that human immortalized epidermal cell migration and proliferation might be induced by ADMSC-exosomes via Wnt/β-catenin signaling. These suggest that MSC exosomes have the ability to quicken the re-epithelization process during the period of proliferation [65]. Table 1 shows some of the lipid categories in exosomes [66–76].

**Table 1:** Lipid categories in exosomes

**Characterization of lipid classes**	**Lipid-related enzymes**	**Functional effects**	**Reference**
SM	–	Triggering calcium influx	[66]
PS	–	Being involved in exosome fate	[67]
Bis (monoacylglycerol) phosphate	–	Multivesicular body formation and subsequent intraluminal vesicle biogenesis	[68]
Cholesterol	–	Regulating exosome secretion	[69]
Ceramides	nSMase2	Sorting cargo into multivesicular bodies	[70]
–	sPLA2 IIA , sPLA2 V	Prostaglandin biosynthesis	[71]
Arachidonic acid, LPC	cPLA2 , iPLA2	Taking membrane curvature into account	[72]
LTA4, LTB4 l, LTC4	LTA4 hydrolase, LTC4 synthase	Mobilization of polymorphonuclear leukocytes	[73]
PGE2	PGE synthase	Inflammation	[74]
PGE2, 15d-PGJ2	COX-1 , COX-2	Immune suppression, PPARγ ligand	[75]
Phosphatidic acid	PLD2 , DGK	Increasing exosome production	[75,76]

*SM* sphingomyelin, *PS* phosphatidylserine, *LPC* lysophosphatidylcholine, *LT* Leukotriene, *PGE2* prostaglandin E2, *15d-PGJ2* 15-Deoxy-∆-12,14-prostaglandin J2, *nSMase* neutral sphingomyelinase 2, *sPLA IIA* secretory phospholipase A2 IIA, *cPLA2* cytosolic phospholipase A2, *iPLA2* anti-phospholipase A2, *COX-1* cyclooxygenase,* PLD2* phospholipase D2, *DGK* diacylglycerol kinase

Exosomes have been linked to numerous important physiological and pathological phenomena, including the elimination of redundant proteins, the presentation of antigens, the transmission of genetic material, immune responses, angiogenesis, inflammation, tumor metastasis, and the dissemination of pathogens or oncogenes [77]. These roles are highly congruent with those of the cells from which they are derived. The components included within exosomes are essential for the aforementioned activities [78]. Exosomes are a kind of EV that are tiny in size and contain a wide variety of biomolecules. Proteins, nucleic acids like DNA, miRNA, lncRNA, mRNA, tRNA and circRNA, and lipids like cholesterol all fall within this category. Surface indicators including CD63, CD81, CD9, tumor susceptibility gene 101 protein (TSG101), heat shock 70 kDa protein (HSP70) and heat shock 90 kDa protein (HSP90) define exosomes, which have a diameter of 30 to 150 nm [79].

The biologically active components of exosome cargo have been observed to facilitate cellular crosstalk when co-incubated with recipient cells or delivered to experimental animals *in vivo*, as evidenced by isolated exosomes. This has been reported in previous studies [80]. Exosomes exhibit heterogeneity in their molecular and genetic composition, which is attributed to their cellular origin. Additionally, exosomes possess the ability to selectively target recipient cells based on specific molecular markers, as directed by the parent cell. Exosomes are known to transmit signals to recipient cells, which ultimately lead to the reprogramming of the cells, whether they are located locally or at a distance [80]. Although the exact mechanisms underpinning the transport and processing of exosome cargo in recipient cells are not yet fully understood, they may begin with the binding of ligands to cell surface receptors, then continue with the exosome’s endocytosis or phagocytosis [81]. By internalizing and delivering nucleic acids, or by signaling via cognate receptors on the exosome surface, exosomes can cause either a loss of function or an increase in function in the receiving cell. This is supported by previous studies [82].

Studies have revealed the presence of ~1600 proteins in exosomes derived from biofluids. Out of these, 300 proteins were found to be common in at least two groups, while only two proteins were shared by more than four groups, as reported in [55].

According to recent research, a total of 860 proteins have been detected, with ~ 110 of them being present in two or more groups. Furthermore, six proteins have been found to be shared by more than five groups, as reported in [83]. The presence of a significant quantity of membrane proteins is apparent among these ubiquitous proteins, including those found in the cytoplasm. Tetraspanins are primarily linked with exosomes. The proteomic investigations also suggest potential physiological functions of exosomes. Exosomal proteins discharged by cultured human keratinocytes have the potential to act as factors that modulate the ECM of dermal fibroblasts, as evidenced by previous research [19]. According to estimates, exosomes, despite constituting a minor fraction of the overall plasma proteome, contain a plethora of proteins that have been modified under diverse pathological circumstances [84].

Following the application of proteomics detection techniques to MSC-exosomes and subsequent pathway enrichment analysis, it was determined that the proteins present in exosomes were primarily associated with functions such as heparin binding, phospholipid binding, integrin, immune response and cell adhesion [69].

Exosomes have the capability to transport mRNAs and miRNAs, which can potentially modify the destiny of the cells that receive them, in addition to their protein delivery function. Upon uptake by human brain endothelial cells of exosomes derived from glioblastoma cells, mRNA molecules are translated and subsequent tubule formation is stimulated [70]. The investigation of mRNA levels has been conducted on exosomes that originate from human breast milk. miRNAs that have been linked to immune regulatory functions exhibit elevated expression during the initial half-year of lactation, but experience a notable decline during subsequent stages [85,86].

According to existing knowledge, exosomes derived from breast milk are believed to play a role in regulating the development of the immune system in infants [87]. Moreover, exosomes possess the capability to disseminate pathogens from one cell to another, as evidenced by the transfer of miRNAs from Epstein Barr virus-infected cells to uninfected recipient cells via exosomes [88]. Exosomes that are secreted from tumor cells have the potential to disseminate oncogenes [89]. This results in the transmission of oncogenic properties to recipient cells, thereby serving as a crucial element in the process of tumor metastasis. In addition to their known functions, it has been suggested that RNAs present in exosomes could serve as potential diagnostic indicators for a range of medical conditions. The identification of distinct expression patterns of serum miRNAs in lung cancer, colorectal cancer and diabetes has demonstrated the potential of serum miRNAs as biomarkers for the detection of diverse diseases, including cancers [90].

Furthermore, the utilization of deep sequencing was employed to gain a more comprehensive understanding of the expression and profiles of miRNAs within exosomes derived from human MSCs. The let-7 family of MSCs derived from human embryos has been observed to have a broad distribution and is linked to the self-renewal properties of stem cells. Recent studies have investigated novel therapeutic targets and strategies for managing acute leukemia through comparative analysis of miRNA expression variations in exosomes obtained from bone marrow MSCs of individuals with acute leukemia and those of healthy individuals [91].

In addition to sequencing miRNAs, the researchers used network analysis to learn more about the genes and pathways that these molecules regulate. Genes targeted by miRNAs were shown to have strong ties to heart repair, regeneration and angiogenesis. Wnt signaling, pro-fibrotic signaling through TGF-stimulation, platelet-derived growth factor (PDGF), proliferation and apoptosis are major pathways involved in this process [92].

Exosomes are found to be abundant with specific raft-associated lipids, including cholesterol, ceramide and phosphoglycerates, as well as long and saturated fatty-acyl chains, in addition to proteins and RNAs. There exist indications that exosomes have the capability to transport prostaglandins to specific target cells [93].

Exosomes derived from MSCs were initially studied in 2010 using a mouse model of myocardial ischemia/reperfusion injury [63]. Subsequently, their efficacy was evaluated in various disease models. According to research, it has been demonstrated that MSCs are capable of generating a greater quantity of exosomes in comparison to other cell types, including myoblasts, the human acute monocytic leukemia cell line (THP-1) and the human embryonic kidney cell line [94], among others [95]. Exosomes derived from MSCs exhibit no discernible distinctions in their morphological characteristics, isolation techniques and storage requirements when compared with those derived from alternative sources. Regarding identification, exosomes derived from MSCs express both typical exosomal surface markers, such as CD9 and CD81, as well as certain adhesion molecules, namely CD29, CD44 and CD73, which are present on the MSC membrane. As with exosomes derived from other sources, the protein constituents of exosomes derived from MSCs exhibit variability when isolated from the conditioned media (CM) of different MSC batches. The liquid chromatography–mass spectrometry/mass spectrometry technique was employed to detect unique proteins in three distinct batches of exosomes derived from MSCs. The results indicate that 379, 432 and 420 unique proteins were detected, with only 154 proteins being common among them [45]. The functional clustering of these proteins indicates that exosomes possess the capability to facilitate numerous biological processes. This concept aligns with the documented effectiveness of MSCs in managing various medical conditions. The presence of proteasome subunits in exosomes derived from MSCs has been reported in the literature [96]. The study reveals a decrease in the buildup of misfolded proteins or oligomers in the cardiac tissues of a murine model of myocardial ischemia/reperfusion injury subsequent to the administration of exosomes derived from MSCs. Moreover, the complete set of proteins present in a 20S proteasome has been identified with a high level of certainty through the utilization of mass spectrometry in the analysis of exosomes derived from MSCs. The degradation of intracellular oxidatively damaged proteins, which may contribute to the cardioprotective activity of MSC-derived exosomes, is attributed to the 20S proteasome. This has been reported in the literature [97]. An investigation of miRNAs present in exosomes derived from MSCs was also conducted. The study has revealed that the miRNAs that are enclosed within microparticles derived from MSCs are primarily present in their precursor state [83]. The microparticles in question exhibit a hydrodynamic radius of 55–65 nm, indicating that they may be classified as exosomes. MSCs have the potential to induce various biological effects in neighboring cells via the secretion of miRNAs in exosomes. The complete abolition of the renal protective effect of MSC-CM is observed upon pretreatment with RNase [44]. The administration of MSC-derived exosomes to neurons and astrocytes results in the upregulation of miR-133b within these cells, thereby facilitating the restoration of function in individuals with Parkinson’s disease and spinal cord injury. The aforementioned discovery implies that MSCs are involved in the regulation of neurite outgrowth through the transmission of miR-133b to neurons and astrocytes by means of exosome secretion [98]. Details of microvesicles and exosomes are given in Table 2 [99–106].

**Table 2:** Micro vesicles and exosomes

**Characteristics**	**Apoptotic body**	**Microvesicle**	**Exosome**	**Reference**
Markers	Positive for permeability in membranes (PI) Chromatin, DNA, and Annexin V	Having a tight membrane (a negative PI test) flotillin-2, selectin and integrin	CD63, TSG101, Alix and flottilin are all PI-negative membrane-impermeable proteins	[99]
Indicator of controlled content	The cellular origin and stimuli	Direct correlations do not exist	Origins and physiological states of cells	[100]
Lipids	Externalization of phosphatidylserine characterizes this species	Plasma membrane lipids provide the primary source of lipids, and the lipid content resembles that of parental cells (without BMP)	Lipid molecules are sorted out of parental cells (including BMPs)	[101]
Density	1.16–1.28 g/ml	1.13–1.19 g/ml	1.13–1.19 g/ml	[102]
Size	Heterogeneous 1–5 μm	Heterogeneous 100–1000 nm	Homologous 30–100 nm	[103]
Mechanism of release	Rho-associated kinase I and myosin ATPase activity	Membrane curvature, phospholipid relocation, cytoskeleton rearrangements, and vesicle release are all involved in the process	The type of cellular origin determines whether it is constitutive or induced	[104]
Detection methods	Flow cytometry, electron microscopy	Flow cytometry, electron microscopy	Exosome-enriched marker western blotting and electron microscopy	[88]
Isolation methods	Ultracentrifugation (10,000–20,000 × *g*)	No standardized methods	Filtration using density gradient and ultracentrifugation (100,000–200,000 g). Exo-Quick precipitation, immunoprecipitation and immune affinity capture	[88]
Identifying and quantifying sizes			Surface plasmon resonance, nanoparticle tracking and dynamic light scattering	[105]
Origin	Apoptosis-related plasma membrane blebbing and cellular debris	Plasma membrane protrusions that grow directly outward	The joining of multicellular vesicles to a plasma membrane	[106]

#### Assimilation of exosomes

The investigation of a particular target for exosomes is presently underway. The mechanism responsible for the targeting of exosomes is commonly attributed to the processes of micropinocytosis and protein, sugar and lipid docking. The exosomes are internalized by the recipient cell and subsequently released through its endosomes [107].

#### Organizing cargoes into exosomes and packaging them

Exosomes contain nucleic acids, proteins and lipids. Exosomes employ a distinct technique for the arrangement and encapsulation of cargoes. Exosomes selectively incorporate exosomal miRNAs and proteins based on the specific cell type and cellular conditions [91]. Exosomes contain miRNA with a conserved GGAG motif known as an EXOmotif, which is not present in cytosolic cell-packaged miRNA. Exosomes are known to contain miRNAs that are packaged through the involvement of sumoylated heterogeneous nuclear riboprotein A2B1 [57]. In addition to ESCRT and tertraspanins, proteins are stored in lipid-dependent compartments [108]. Exosomes exhibit a higher concentration of cholesterol, sphingomyelin, saturated phosphatidylcholine and phosphatidylethanolamine in comparison to the plasma membrane of the cell [109].

Exosomes are known to contain a variety of biomolecules such as RNA, proteins, lipids and metabolites, which can provide insights into the cellular origin of these extracellular vesicles. Exosomes are frequently subjected to extensive proteomics and transcriptomics analyses owing to their elevated protein, RNA and lipid composition [110]. FunRich is a software application that is not intended for commercial purposes and can be utilized for the purpose of identifying over-represented clusters of molecules from a given dataset. The advancement of next-generation sequencing technologies has expedited the study of exosomes, in addition to cancer research. A recent study has revealed that there is an association between exosomes derived from *Trypanosoma cruzi* and a diverse range of significant gene products. This enhances the likelihood of identifying biomarkers for Chagas disease through the utilization of bioinformatics analysis [111].

The potential of exosomes as therapeutics is becoming more evident due to their ability to induce robust cellular responses both *in vitro* and *in vivo*. Exosomes have been found to promote regenerative effects in cases of injury and disease that are similar to the biological activity observed in stem cells. Several signaling pathways, namely Akt, ERK and STAT3, are implicated in the process of wound healing and bone fracture repair [112]. Mesenchymal stem cell-derived extracellular vesicles (MSC-Exos) have been demonstrated to modulate immunity-mediated responses and inflammation. The aforementioned factors are known to induce the expression of various growth factors, including but not limited to hepatocyte growth factor, insulin-like growth factor-1, nerve growth factor [89] and stromal-derived growth factor-1 [113]. The proangiogenic properties of human circulating fibrocyte exosomes were observed *in vitro*. Additionally, these exosomes were found to activate diabetic dermal fibroblasts, stimulate the migration and proliferation of diabetic keratinocytes and accelerate wound closure in diabetic mice. Mesenchymal progenitor cells secrete exosomes that have a significant impact on the process of wound healing by means of paracrine signaling [114]. The exosomal cargo primarily consisted of various components, including HSP-90α, both total and activated signal transducers and activators of transcription 3, proangiogenic miRNAs such as miR-126, miR-130a and miR-132, anti-inflammatory miRNAs such as miR-124a and miR-125b, and a miRNA that regulates collagen deposition, namely miR-21 [115]. The administration of human exosomes to rat wounds was observed to be associated with accelerated wound healing, specifically due to the application of oral keratinocyte exosomes. The utilization of exosomes as a delivery mechanism for siRNA has demonstrated a certain level of efficacy, owing to their presence in the endogenous system of the body and their high tolerance. Several clinical trials have utilized exosomes derived from patients as a cancer immunotherapy intervention in experimental settings [116].

Exosomes are considered to be efficient drug carriers as a result of their cellular membranes and multiple adhesive proteins on their surfaces, which enable them to deliver various therapeutic agents to target cells. Exosomes were utilized as a delivery mechanism for the pharmaceutical agent paclitaxel. Resistant lung cancer was induced in mice, and subsequently, exosomes derived from white blood cells containing the drug were administered via injection [74].

### Exosome components implicated in wound infection healing

Wounds or injuries are the result of various forms of physical damage to the skin, such as scratching, tearing, cutting, piercing or trauma. Despite the outward appearance of healthy skin, there may be the underlying presence of red or bruised marks. The term “wound” is commonly used to refer to the disruption of intercellular connections within the skin or flesh. In the realm of medical terminology, wounds are classified into two distinct categories: closed and open. Open wounds encompass injuries such as cuts and skin tears, while closed wounds refer to internal damage such as bruises. The colonization of bacteria in a wound is the primary cause of infection. The occurrence of a wound infection can be attributed to various bacterial strains such *as P. aeruginosa, Escherichia coli*, *Proteus mirabilis*, *Acinetobacter baumannii/haemolyticus*, *Streptococcus* and *S. aureus* [94,108].

Several wound treatment modalities have been suggested, including pharmacotherapy, cellular therapy, extracorporeal shock wave therapy, negative pressure wound therapy, electrical stimulation therapy and light therapy. Despite the implementation of numerous preventative measures, a variety of complications may arise during both exogenous and endogenous processes. Furthermore, the emergence of secondary infections has an adverse impact on the overall health status of the patient, leading to extended hospital stays and recovery periods, and substantially augmenting the expenses associated with medical care [117].

The prevalence of drug-resistant bacteria and diseases such as diabetes mellitus, which predispose to chronic infection, have contributed to the persistence of wound infections as a significant cause of morbidity and mortality in patients [118]. Infections that exhibit the formation of bacterial biofilms, commonly observed on the exterior of implants including catheters, orthopedic devices or partially devitalized tissues, pose a significant challenge for treatment with antibiotics alone. Such infections often necessitate prolonged and continuous therapy spanning several weeks to months [63,96,112,119].

Previous research has established that MSCs possess antimicrobial properties. Both direct and indirect effects have been demonstrated. Studies have demonstrated that MSCs possess the ability to release antimicrobial peptides such as cathelicidins, lipocalin-2 and beta-defensins. Several studies have demonstrated that the secretion of cathelicidin LL-37 by MSCs can be augmented by bacterial products, suggesting that MSCs have the ability to increase their antimicrobial activity in the presence of an infection. It has been shown that MSCs possess the ability to engage with the innate immune system of the host, thereby augmenting the antibacterial activity. Numerous studies have exhibited heightened phagocytic and bactericidal capabilities of monocytes and neutrophils subsequent to exposure to factors secreted by MSCs. Additionally, it has been noted that MSCs possess the ability to mitigate inflammation in models of sepsis [120,121].

ADSC exosomes (ADSC-Exos), a recently developed biotechnology, have demonstrated significant advancements in various domains, particularly in the area of cutaneous wound healing. In contrast to auxiliary maintenance modes observed in certain conventional therapies, ADSC-Exos primarily facilitate wound healing by stimulating the activation of wound-healing mechanisms [122]. The process of skin wound repair involves a sequence of cellular interactions and reactions with various mediators. Despite the fact that there is still a significant amount of information yet to be discovered regarding the fundamental mechanisms of skin repair, a growing number of *in vivo* and *in vitro* experimental studies are revealing the effectiveness and safety of ADSC-Exos in the process of skin repair [123]. In the context of ischemia, inflammation represents the initial response among the four classic wound repair mechanisms. The initial stages of wound healing are marked by a range of inflammatory reactions, such as hyperemia, serous exudation and the infiltration of leukocytes. A moderate inflammatory response is advantageous for tissue repair as it aids in the removal of cell debris, fights infection and eliminates inflammatory factors. Chronic wounds have been found to be linked with an unfavorable inflammatory milieu [112]. Exosomes released by MSCs have been found to play a role in the regulation of inflammatory responses. Exosomes containing proteins and RNA have been observed to exert a notable influence on the regulation of inflammation. MSC-Exos have been found to possess a significantly elevated concentration of miR-223. A study conducted by Wang *et al*. [53] revealed that exosome release was linked to MSC-induced cardio-protection, wherein exosomes derived from WT-MSCs were utilized. The study found that the overexpression of miR-181c in exosomes derived from human umbilical cord mesenchymal stem cells (hUCMSC) led to a reduction in inflammation in rats with burn injuries. This was achieved through the downregulation of the LPS-induced TLR4 signaling pathway. The administration of MSC-Exos via intravenous injection in a model of dilated cardiomyopathy was observed to have a mitigating effect on the activation of macrophages, which was previously mediated by the JAK2-STAT6 pathway. Under specific preconditioning conditions, MSCs exhibit a noteworthy enhancement in their paracrine effect, leading to a reduction in the expression of inflammatory factors and an improvement in the inflammatory microenvironment. The study examined the efficacy of LPS preExos, derived from MSCs, in addressing chronic inflammation and wound healing. The findings indicated that LPS preExos exhibited superior anti-inflammatory properties compared to un-Exos. The LPS preExos facilitated macrophage polarization and resolved chronic inflammation by interacting with the let7b/TLR4 pathway. Furthermore, the transfer of miR-146a via exosomes, which is a widely recognized anti-inflammatory miRNA, resulted in enhanced immunomodulatory effectiveness of hUCMSCs that had been pre-treated with IL-1β) [87]. The aforementioned discoveries present novel avenues for further investigation and practical implementation of exosomes within this domain [63]. MSCs and ADSCs exhibit comparable attributes. Several studies have shown the efficacy of allogeneic adipose tissue-derived mesenchymal stem cell conditioning media (ADSC-CM) in restoring inflammation balance in immunocompromised obese individuals. It was found that ADSC-CM was effective in counteracting persistent inflammation in the study. ADSC-Exos are also capable of reducing the production of inflammatory mediators, thus alleviating the effects of osteoarthritis. ADSC-Exos inhibit senescence-associated galactosidase activity, which is a significant factor contributing to the development of osteoarthritis. In spite of the lack of available evidence indicating that skin wounds can be healed, the results presented here suggest a potential treatment avenue for ADSC-Exos [37,48,49].

### Wound angiogenesis

Wound angiogenesis plays a critical role in the process of wound healing. During the wound healing process, granulation tissue is formed as slender-walled capillary buds develop and fibroblasts proliferate. This formation is encapsulated or organized by necrosis, thrombosis, inflammatory exudates, and other foreign bodies. This results in the formation of a matrix that facilitates the migration of keratinocytes [124]. Sufficient vascularization is essential for the successful regeneration of healthy tissue, as it facilitates the provision of vital nutrients, oxygen and pathways for cellular migration [125]. Angiogenesis is dependent on ADSCs. Exosomes are mainly responsible for ADSC angiogenesis. There is evidence that the most conserved ASC secretome proteins are detected when differentiating or pro-inflammatory stimuli are present in exosome studies [126]. Cell stimulation controls the protein content of exosomes, depending on the original cells. The angiogenic stimulators vascular endothelial growth factor A and FGF2, especially b-FGF, are well acknowledged. Overexpression of nuclear factor-E2-related factor 2 by ADSC-Exos decreased ulcerated regions in a rat model of diabetic foot ulcers and promoted endothelial progenitor cell proliferation and angiogenesis. This provides supporting evidence for the therapeutic potential of exosomes produced from ADSCs in the management of diabetic foot ulcers. Human microvascular endothelial cells showed the development of a long-lasting vascular-like structure within 48 h of EV stimulation after being exposed to free-EV supernatant or stimulated with adipose-derived EVs following PDGF stimulation *in vitro*. The difference between the experimental and control groups was large enough to be considered statistically significant. Previous studies have suggested that ADSC-derived EVs could improve human microvascular endothelial cells’ angiogenic capacity *in vivo* [127].

The therapeutic mechanism of MSC-Exos in wound angiogenesis involves their ability to promote enhanced vascularization and angiogenesis, which are essential for the wound healing process. MSC-Exos have been shown to induce angiogenesis through the secretion of pro-angiogenic factors, such as vascular endothelial growth factor, FGF-2, hepatocyte growth factor and PDGF-BB. Additionally, MSC-Exos have been reported to enhance angiogenesis via the AKT/endothelial nitric oxide synthase pathway, leading to the development of new blood vessels in the wound site. These properties of MSC-Exos make them a promising therapeutic option for promoting wound angiogenesis and accelerating the healing process. As a result of the subcutaneous injection of ADSC EVs into male severe combined immunodeficiency mice, human microvascular endothelial cells were significantly more able to form new blood vessels after a 10-day pre-stimulation period [128]. Human microvascular endothelial cells injected with ASC-EVs enhanced angiogenic potential after pre-stimulation with ASCs for 10 days [129]. The study yielded findings indicating that ASC-EVs are equipped with angiogenic factors, including but not limited to MMPs, MFG-E8, ANGPTL1 and thrombopoietin. The production of EVs is augmented by PDGF, which also regulates the levels of proangiogenic and antiangiogenic factors present in EVs that are discharged from ASCs. These EVs are responsible for promoting angiogenic activity, as per the findings of a previous study [83]. The miRNAs found in ASC-released MVs have been observed to participate in the proangiogenic process in human umbilical vein endothelial cells [130]. Angiogenesis, the process by which new blood vessels are formed, may be stimulated by using MVs generated by stem cells, as shown in [65]. miR-125a was shown to be concentrated in ADSC-Exos and then transported to endothelial cells, as reported by Liang *et al*. Delta-like 4 expression was suppressed as a consequence and angiogenesis was modulated by promoting endothelial tip development [131]. IL-6 levels exhibited an elevation in skin flaps sourced from ADSC-Exos in the context of ischemia/reperfusion [132]. Furthermore, IL-6 facilitates the process of angiogenesis in addition to its role in promoting flap recovery. The findings suggest that ADSC-Exos exhibit potential as a viable option for promoting wound angiogenesis. However, it is imperative to clarify their protein composition [133].

### Wound proliferation

Cellular proliferation, migration, proteoglycan synthesis, and the production of type 1 and type 3 procollagen are essential characteristics of wound healing. During this stage, the formation of new tissue occurs [134]. Matrix proteins facilitate cellular attachment and repair by creating an extracellular environment, which enables epithelial cells to migrate from the periphery to the site of injury [135]. During this phase, the synthesis and deposition of the ECM takes place. Re-epithelialization, a significant occurrence in the formation of new tissue, is brought about by an interaction between epithelial and stromal cells. The process of ECM deposition and remodeling is significantly facilitated by skin fibroblasts [136]. Fibroblasts differentiate into myofibroblasts when prompted by other cells. Both normal and diabetic chronic wound fibroblasts had their proliferation and migration boosted by MSC-Exos, and this effect was found to be dosage dependent. In contrast, MSC-CM deficient in exosomes did not show the same impact [137]. The connection between ASC exosomes and wound healing was investigated by Hu *et al*. in a rat model. Labeled ADSCs-Exos may be taken up by fibroblasts, as shown by *in vitro* tracking tests. Protein expression of N-cadherin, cyclin-1, PCNA, collagen I and III, and elastin was significantly upregulated after exposure to ADSC-Exos, as determined by Masson’s staining, immunohistochemistry and quantitative RT-PCR. Based on these results, exosomes appear to aid in the earliest phases of the wound-healing process. Recent research suggests that exosomes may impede collagen formation [138]. MSC-Exos play a crucial role in promoting wound proliferation through various mechanisms. These exosomes have been shown to promote enhanced cell migration, proliferation, and migration of epithelial cells and fibroblasts, which are essential for the proliferative phase of wound healing. Additionally, they induce angiogenesis, which further supports tissue proliferation and regeneration. Furthermore, MSC-Exos have been reported to accelerate wound healing at various phases, including the proliferative stage, and have the ability to improve scars. By regulating the different stages of wound healing, MSC-Exos effectively reduce tissue inflammation, promote enhanced cell migration and promote proliferation, thereby contributing to the overall wound healing process. Therefore, the therapeutic mechanism of MSC-Exos in wound proliferation involves their ability to modulate various aspects of the wound healing process, ultimately promoting enhanced cell proliferation, migration and tissue regeneration [14,139]. As per the findings, the administration of intravenous injections is observed to have a greater impact on wound healing in comparison to local injections, which is contrary to the anticipated outcome [140]. The distribution of exosomes was found to be higher in the vicinity of inguinal wounds with an adipose tissue layer as opposed to dorsal wounds lacking such a layer. What is the composition of exosomes? Studies have shown that exosomes that are isolated from ADSC have the ability to induce HDF cell migration and angiogenesis in the context of wound healing in a rat model of ischemic wound healing, as well as in *in vitro* scratch assays and ECIS assays. This phenomenon is found to be dependent on the presence of MALAT1, an alpha-nucleotide RNA that has a significant association with metastasis in patients with lung cancer [49,141]. Therapeutically useful lncRNAs are present in exosomes. Exposure to adipose-derived MSC-EVs increases keratinocyte and fibroblast proliferation, migration and AKT activation, according to research [142]. The study showed that using a gel containing MSC-EVs produced from adipose tissue accelerated wound healing in an animal model. The contents of exocrine bodies are varied and incompletely described [143,144].

### Wound remodeling

The process of ECM remodeling generally spans a duration ranging from 2 weeks to 1 year. The phases in the process of wound remodeling significantly influence the reorganization and production of ECM, which in turn plays a major role in determining scarring [145]. During the advanced phases of wound healing, it has been observed that effector cells undergo apoptosis, collagen III is replaced by collagen I, matrix metalloprotease degradation occurs and the synthesis of other ECM proteins takes place [146]. MSC-Exos play a significant role in wound remodeling through various mechanisms. These exosomes have been shown to accelerate wound healing at different phases, including the remodeling stage, and even have the ability to improve scars. They achieve this by promoting enhanced cell migration, proliferation and angiogenesis, which are essential for tissue remodeling and regeneration. Additionally, MSC-Exos have been reported to have anti-fibrotic effects and to modulate the expression of genes associated with tissue remodeling, ultimately contributing to improved wound healing outcomes. Therefore, the therapeutic mechanism of MSC-Exos in wound remodeling involves their ability to modulate various aspects of the wound healing process, ultimately promoting enhanced tissue regeneration and scar improvement [147,148].

In an experimental environment, ADSC-EVs and bone marrow EVs were injected intradermally into a wound healing model by Pelizzo in 2018 [149]. ADSC-Exos inoculation was followed by the appearance of regenerated connective tissue, intact epithelium, dermal papillae and cutaneous annexes [138]. Intravenous administration of ADSC-Exos was used in a mouse model with a full-thickness dorsal wound. In addition to suppressing fibroblast development into myofibroblasts and granulation tissue formation, ADSC-Exos have been shown to increase the ratio of collagen III to collagen I *in vivo*. Exosomes’ ability to regulate the ECM has been linked to their ability to promote scar-free skin regeneration. Activation of the ERK/MAPK pathway in skin dermal fibroblasts raised the MMP3/TIMP1 ratio [14]. Both *in vitro* and *in vivo* evidence supports the use of ASCs-Exos for the treatment of soft tissue wounds. Across the board in wound healing, ADSC-Exos demonstrate a synergistic and complimentary role. To determine the efficacy of ADSC-Exos, it is necessary to undertake large-scale prospective, randomized, blinded and placebo-controlled clinical studies [150–152].

### Exosome components implicated in bacterial wound infection

Wound healing with exosomes produced by MSCs is an area that shows great potential. The possible advantages of modified MSC-Exos in the area of wound healing are the focus of current research. Recent studies have shown that MSC-Exos may help speed up the healing process for skin wounds by increasing the production of new blood vessels, facilitating the migration of existing ones, and stimulating the growth of both acute and chronic wounds. There is promising evidence that loading MSC-Exos with biomaterials can increase concentration, bring about the intended therapeutic benefits and guarantee a longer release [87,153]. The efficacy of MSC-Exos in wound healing has been well-documented, and MSC-Exos engineered through bioengineering show promise as a wound treatment. Research has shown that bioengineered MSC-Exos can improve the healing and regeneration of skin lesions, for instance. Discoveries have been made about the ability of exosomes produced by MSCs to control the inflammatory response to wounds and expedite their recovery. BMSCs and ADSCs are two other types of stem cells that can aid in wound healing. In addition to skin and epidermal wounds, MSC-Exos have been used for wound healing in other organs as well. Continuous research is being conducted to enhance the therapeutic application of MSC-Exos, which have great promise for personalized and regenerative medicine [154,155].

MSC treatment improves neutrophil migration and phagocytic activities, which leads to better bacterial clearance. The stimulation of cytokines including IL-6, IL-8 and granulocyte-macrophage colony-stimulating factor accomplishes this. Together, these chemicals eradicate the infection and facilitate tissue regeneration, as seen by the healing of wounds [156].

As mentioned before, skin wound healing is a complex process that includes several stages, including inflammation, remodeling, proliferation and homeostasis. It is well recognized that MSCs contribute to every stage of the wound-healing process, offering potential therapeutic advantages [157].

Antimicrobial peptides (AMPs) can be produced by MSCs as well. AMPs either destroy bacteria directly by rupturing their membranes or indirectly by causing the production of cytokines that promote inflammation. MSCs secrete a range of AMPs, including lipocalin 2, β-defensin 2, hepcidin and the cathelicidin peptide LL-37 [158]. These AMPs are thought to be essential modulators of MSCs’ therapeutic potential to eradicate bacterial infections. MSCs can secrete PGE2, IL-6, IL-8 and IFN-β, among other things, which have the direct effect of influencing the immunological characteristics of neutrophils and macrophages [159].

Additionally, the antimicrobial properties of exosomes generated by MSCs accelerate the healing of diabetic foot ulcers. Exosomes contain both inert and physiologically active components, including proteins, growth factors and nucleic acids. MSC-exosomes reduce the elimination of microorganisms by augmenting neutrophil migration and phagocytic potential via increased levels of IL-6, IL-8 and granulocyte-macrophage colony-stimulating factor. As the wound-healing process demonstrates, these chemicals eventually aid in the removal of the infection and encourage tissue regeneration [87,160].

Additionally, MSCs are essential for controlling the immune system and eliminating harmful microbes. A number of notable AMPs, including as LL-37, β-defensin-2, cathelicidin, hepcidin and lipocalin-2, are known to be produced by MSCs. These peptides have an impact on MSC migration, proliferation control and regeneration activities. In a similar vein, MSCs overexpress IL-17 and indoleamine-2,3-dioxygenase. Strong antimicrobial action of indoleamine-2,3-dioxygenase is demonstrated against a wide range of pathogens, including viruses (cytomegalovirus, herpes simplex virus), bacteria (*S. aureus*, *Staphylococcus epidermidis*, group B streptococci and *Enterococcus faecium*) and parasite infections (Toxoplasma gondii). According to the findings of an investigation, MSCs can increase the antimicrobial potential of horse keratocytes by encouraging AMP expression through CCL2 production [161].

As a result of their immunomodulatory and anti-inflammatory properties, ADSC-Exos play an important role in preventing local inflammation and necrosis [130]. ADSC-Exos suppress T cell proliferation and inflammatory factor secretion by blocking the differentiation and activation of T cells. According to the results of several studies, ADSC-Exos are in charge of controlling the phenotypic polarization of macrophages, which is how they bring about a decrease in adipose inflammation and obesity [128]. Furthermore, it is noteworthy that ADSC-Exos are comprised of miRNAs, which are a class of small, endogenous, non-coding nucleotides that play a regulatory role in metabolism and growth. According to a recent study, miRNA-451a was discovered to be enriched in ADSC-Exos, leading to the downregulation of macrophage migration inhibitory factor (MIF) and promoting macrophage polarization between M1 and M2 [156]. MIF is a pro-inflammatory mediator that controls immunity *in vivo* and has a wide range of effects. IL-6 and tumor necrosis factor alpha are two inflammatory mediators that are suppressed when MIF levels are raised. Improvements in arthritis and cartilage degradation can be achieved by decreasing inflammation. The immune-modulatory proteins retinol-binding protein 4 and macrophage colony-stimulating factor are also produced by ADSC-Exos [162]. As soon as cells are destroyed by macrophages, proteolytic enzymes are released, which in turn degrade necrotic tissue and debris, facilitating the healing of wounds. Specifically, ADSC-Exos appear to aid wound healing in diabetic foot ulcer rats overexpressing nuclear factor-E2-related factor 2, and oxidative stress-related inflammatory cytokines and proteins are reduced [163]. ADSC-Exos exhibit analogous characteristics to those of their progenitors. They upregulate inflammation and angiogenesis, thereby enhancing graft retention. In addition to inducing pro-inflammatory macrophage inflammation, ADSC-Exos upregulate the expression of monocyte chemoattractant protein-1 and macrophage inflammatory protein-1. Further research is required to investigate the potential of ADSC-Exos in modulating immune function and mitigating inflammation in chronic wounds [84].

## Conclusions

The chronic wound healing process entails a multitude of factors that operate in a closely coordinated series of steps. ADSC-Exos exhibit promising potential as a therapeutic intervention for chronic wounds due to their ability to mitigate oxidative stress, promote neovascularization, induce collagen deposition and minimize scarring. Based on the findings in this particular area of study, it is anticipated that ADSC-Exos may have the potential for treating a broad spectrum of illnesses, thereby serving as therapeutic agents for various maladies in the future.
